# CT findings and clinical effects of high grade pancreatic intraepithelial neoplasia in patients with intraductal papillary mucinous neoplasms

**DOI:** 10.1371/journal.pone.0298278

**Published:** 2024-04-29

**Authors:** Min Cheol Kim, Jung Hoon Kim, Sun Kyung Jeon, Hyo-Jin Kang

**Affiliations:** 1 Department of Radiology, Seoul National University Hospital, Seoul, South Korea; 2 Department of Radiology, College of Medicine, Seoul National University, Seoul, South Korea; 3 Institute of Radiation Medicine, Medical Research Center, Seoul National University, Seoul, South Korea; National Cancer Institute, UNITED STATES

## Abstract

**Purpose:**

To investigate the common CT findings of high-grade **(**HG) PanIN and clinical effects in the remnant pancreas in patients with intraductal papillary mucinous neoplasm (IPMN) of the pancreas.

**Materials and methods:**

Two hundred fifty-one patients with surgically confirmed IPMNs (118 malignant [invasive carcinoma/high-grade dysplasia] and 133 benign [low-grade dysplasia]) were retrospectively enrolled. The grade of PanIN (233 absent/low-grade and 18 high-grade) was recorded, and all patients underwent serial CT follow-up before and after surgery. Two radiologists analyzed CT findings of high-risk stigmata or worrisome features according to 2017 international consensus guidelines. They also analyzed tumor recurrence on serial follow-up CT after surgery. Statistical analyses were performed to identify significant predictors and clinical impact on postoperative outcomes of HG PanIN.

**Results:**

PanIN grade showed a significant association with IPMN grade (p = 0.012). Enhancing mural nodules ≥5 mm, abrupt main pancreatic duct (MPD) changes with distal pancreatic atrophy, increased mural nodule size and MPD diameter were common findings in HG PanIN (P<0.05). In multivariate analysis, abrupt MPD change with distal pancreatic atrophy (odds ratio (OR) 6.59, 95% CI: 2.32–18.72, <0.001) and mural nodule size (OR, 1.05; 95% CI, 1.02–1.08, 0.004) were important predictors for HG PanIN. During postoperative follow-up, HG PanIN (OR, 4.98; 95% CI, 1.22–20.33, 0.025) was significantly associated with cancer recurrence in the remnant pancreas.

**Conclusion:**

CT can be useful for predicting HG PanIN using common features, such as abrupt MPD changes and mural nodules. In HG PanIN, extra caution is needed to monitor postoperative recurrence during follow-up.

## Introduction

Pancreatic intraepithelial neoplasia (PanIN) is a well-known precursor to pancreatic ductal adenocarcinoma (PDAC) [1]. PanIN is a microscopic, flat or papillary epithelial neoplasm arising in the small intralobular pancreatic ducts usually less than 5 mm in size. It is considered the most important precursor of PDAC in a stepwise carcinogenesis model from intraepithelial neoplasia to invasive carcinoma. Based on the degree of cytological and architectural atypia, PanINs are classified into two grades: PanIN-1 (low-grade dysplasia) and PanIN-2 (moderate dysplasia) are categorized as low-grade PanIN (LG PanIN) and PanIN-3 (carcinoma in situ or high-grade dysplasia) is categorized as high-grade PanIN (HG PanIN) [1, 2]. While intraductal papillary mucinous neoplasm (IPMN) and mucinous cystic neoplasm (MCN), less frequent precursors of PDAC, appear as cystic lesions on imaging studies and can be diagnosed before surgery, PanIN is a microscopic lesion that is almost impossible to detect macroscopically, and is typically found pathologically to be accompanied by surrounding invasive cancer after pancreatic resection [3].

Therefore, although PanIN is the most common precursor of PDAC, the imaging findings of PanIN or the clinical significance of PanIN identified after surgery has been insufficiently considered. Recently, several previous publications have attempted to diagnose HG PanIN using imaging [4, 5]. Because HG PanIN without invasive carcinoma does not form a gross mass, most of the studies presented indirect imaging findings for the detection of HG PanIN in association with its histopathological features. However, most of the preceding studies are based on a small number of subjects and focused mainly on endoscopic ultrasound (EUS) and magnetic resonance imaging (MRI); few studies have emphasized the role of computed tomography (CT) for diagnosing HG PanIN. However, as CT is routinely used as the primary imaging modality for preoperative examination and treatment monitoring in patients with pancreatic cancer, determining the features that can depict HG PanIN on CT is important [6].

Although the presence of PanINs and their grades found after pancreatectomy of IPMN of the pancreas have been presumed to be related to the prognosis, no significant association has been demonstrated in previous studies [7–10]. Considering that high-grade ductal dysplasia accompanying IPMN, another precursor of pancreatic cancer, is significantly associated with the risk of developing invasive cancer and recurrence after surgery, further studies are still needed on the potential association between the presence of HG PanIN after pancreatectomy and the risk of recurrence of pancreatic cancer [11]. Thus, the purpose of this study is to investigate the common CT findings of HG PanIN and its clinical effect in the remnant pancreas after surgical resection of IPMN of the pancreas.

## Materials and methods

This retrospective study was approved by the institutional review board of Seoul National University Hospital. The requirement for informed consent was waived. (No 2007-183-1143). All methods were performed in accordance with the relevant guidelines and the principles expressed in the Declaration of Helsinki.

### Patients

**Fig 1:**
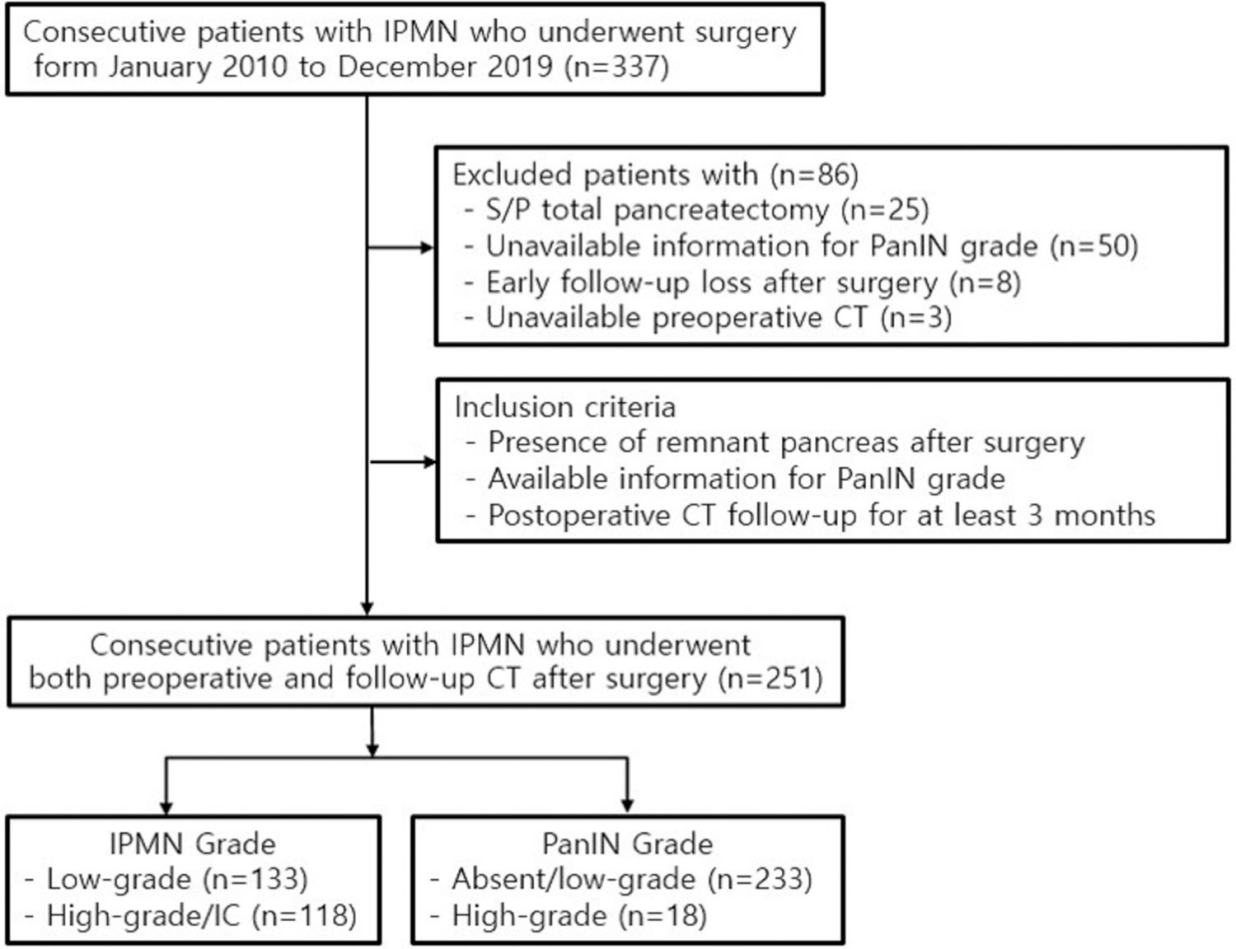
A flow chart of patient enrolment. IPMN = intraductal papillary mucinous neoplasm, PanIN = pancreatic intraepithelial neoplasia, CT = computed tomography, S/P = status post.

From January 2010 to December 2019, we searched a list of 337 consecutive patients with pathologically proven IPMN who underwent pancreatectomy using a text search function in the picture archiving and communication system and electronic medical records of our institution. These data were accessed for research purposes on January 2021. To evaluate pre- and postoperative imaging findings throughout the patients’ clinical course, the following inclusion criteria were used: (1) patients with remnant pancreas after partial pancreatectomy, (2) patients with a documented grade of PanIN at the time of surgery, and (3) patients who underwent postoperative CT follow-up for at least 3 months. Of the 337 patients, 86 were excluded for the following reasons: no remnant pancreas due to total pancreatectomy (n = 25), unavailable record of PanIN grade (n = 50), early follow-up loss within 3 months after surgery (n = 8), and unavailable preoperative CT (n = 3). As a result, 251 patients (150 men, 101 women; mean age, 72.1 years; range, 41–91 years) were finally enrolled in this retrospective study. The types of surgeries were the Whipple operation or pylorus-preserving pancreaticoduodenectomy (n = 146), distal pancreatectomy (n = 94), and central pancreatectomy (n = 11). **Fig 1** presents a flowchart of the patient enrolment process.

### CT acquisition

All patients underwent a total of 251 preoperative CT examinations using one of the various multidetector-row CT scanners. The details of the CT scanners and acquisition parameters are provided in the (**S1 Table and S1 File**). Each patient underwent a variable number of serial postoperative follow-up CT scans for postoperative surveillance. The mean interval between preoperative CT and surgery was 23 days (range, 0–254 days). The mean interval between surgery and the last follow-up CT was 1300 days (range, 96–4108 days).

### CT image interpretation

Two abdominal radiologists (H.J.K. and S.K.J., with 10 and 8 years of experience in abdominal imaging, respectively) reviewed all CT images independently. All readers were informed that the patients had pathologically proven IPMN by surgery but were blinded to the patients’ IPMN or PanIN grade confirmed by surgery and their clinical course during the follow-up period.

To assess common imaging features of HG PanIN on preoperative CT, each reviewer assessed whether IPMNs on preoperative CT demonstrated any of the high-risk stigmata or worrisome features, based on image parameters of the revised 2017 international consensus guidelines (**S2 Table**) [12]. Other analyzed items included the location of pancreatic involvement, multiplicity, and type of IPMN (main duct, branch duct or mixed type). The size of the pancreatic cystic neoplasm was measured as the longest diameter in any axial, coronal or sagittal image. The diameter of the MPD was measured at the maximum point of pancreatic duct dilation. Enhancing mural nodules were recorded as present when there were any papillary protruding lesions that showed contrast enhancement within the cyst or dilated duct. A thickened and enhancing cyst wall was defined as a cyst wall with a width of at least 2 mm and contrast enhancement. Enlarged peripancreatic lymph nodes with a maximum short diameter exceeding 10 mm were considered lymphadenopathies. All measurements were made on either axial, coronal or sagittal images. The cyst growth rate was not evaluated in this study because only findings from a single CT examination before surgery were analyzed. In patients with multiple identifiable pancreatic cysts, the lesion with the highest risk was analyzed. If the lesions showed none of the high-risk stigmata or worrisome features, the largest lesion was analyzed. In case of disagreement, additional review of the unmatched items by a third radiologist (J.H.K, with 24 years of experience in abdominal imaging) was performed to reach consensus.

To evaluate patients’ postoperative outcomes, tumor recurrence, new cystic neoplasm formation, an increase in cyst size and MPD dilatation were assessed on serial follow-up CT. An increase in cyst size was defined as an increase of at least 20% and 2 mm in the longest diameter of the preexisting cyst. MPD dilatation was regarded as present when the MPD diameter was at least 5 mm and was recorded in two categories: 5–9 mm and 10 mm or more.

### Pathologic data collection

Information on the World Health Organization (WHO) grades of IPMN and PanIN was collected from the pathologic reports. The WHO grade of IPMN was classified as low-grade dysplasia, high-grade dysplasia, or associated invasive carcinoma [13]. In this study, IPMNs with low-grade dysplasia were classified as benign IPMNs, whereas IPMNs with high-grade dysplasia and associated invasive carcinoma were categorized as malignant IPMNs, as described in previous studies [14, 15]. Moreover, PanINs with WHO grades 1A, 1B, and 2 were classified as LG PanINs, whereas PanINs with WHO grade 3 were regarded as HG PanINs [16].

### Recurrence analysis

All patients were treated with a standardized protocol that included follow-up contrast-enhanced CT or MR imaging every 3–6 months with serum carbohydrate antigen (CA) 19–9 measurement to assess tumor recurrence after surgery. In our institution, patients with IPMN generally undergo postoperative follow-up CT or MR imaging every 3–6 months, either alone or alternately. Subsequently, surveillance intervals or durations are modified based on the individual patient’s risk factors and other relevant considerations. Tumor recurrence was defined as any newly developed enhancing soft tissue lesion in the remnant pancreas or resection margin that grew in size on follow-up imaging.

### Statistical analysis

The linear-by-linear association was used to analyze the relationship between IPMN grades and PanIN grades. The preoperative CT findings were compared between benign and malignant IPMNs and then between absent/low-grade and HG PanINs using the chi-square test or Fisher’s exact test for categorical variables and Student’s t test for continuous variables. Subsequently, logistic regression analyses with the forward projection method were performed to identify significant predictors for malignant IPMN and HG PanIN. First, univariate analysis was performed for each image finding, and only variables with a p value of < 0.05 were selected as input variables for multivariate analysis. The optimal cutoff value was set using receiver operating characteristic (ROC) analysis and the Youden index. The postoperative follow-up CT findings were also compared between benign and malignant IPMNs and then between absent/low-grade and HG PanINs using the same statistical methods. To assess the interreader reliability of CT findings, the Cohen κ values were derived and interpreted as follows: 0.81–1.00, excellent agreement; 0.61–0.80, good agreement; 0.41–0.60, moderate agreement; 0.21–0.40, fair agreement; and 0.00–0.20, poor agreement. All statistical analyses were performed using SPSS Statistics for Windows, version 25.0 (IBM Corp.). P values less than 0.05 were considered statistically significant

## Results

Patient demographics and clinical characteristics are shown in **Table 1**. Of 251 patients, 118 were confirmed to have malignant IPMNs (69 with invasive carcinoma and 49 with high-grade dysplasia) and 133 were confirmed to have benign IPMNs. In total, 18 patients had HG PanINs and 233 patients had absent/LG PanINs (112 with absent, 121 with LG PanIN). PanIN grade showed a significant positive association with IPMN grade (p = 0.012). In terms of multiplicity, the malignant IPMN group showed a significantly higher frequency of a single lesion than the benign IPMN group (p = 0.006). The type of IPMN showed a significant difference between the high-grade and absent/low-grade PanIN groups (p = 0.016). Patient age, sex distribution, location of IPMN, and type of surgery did not significantly differ between the groups (Ps >0.05).

**Table 1:** Summary of study population demographics and clinical characteristics.

Characteristic		Total (n = 251)	IPMN			PanIN		
			Benign (n = 133)	Malignant (n = 118)	P value	Absent/low grade (n = 233)	High grade (n = 18)	P value
Sex (M:F)		150:101	86:47	64:54	0.093	142:91	8:10	0.169
Mean age (range, years)		72.1 (41–91)	71.8 (41–88)	72.1 (41–91)	0.111	72.0 (41–91)	73.4 (60–88)	0.555
Multiplicity	Single	179 (71.3)	85 (63.9)	94 (79.7)	**0.006**	166 (71.2)	13 (72.2)	0.930
	Multiple	72 (28.7)	48 (36.1)	24 (20.3)		67 (28.8)	5 (27.8)	
Location	Head, uncinate	136 (54.2)	67 (50.4)	69 (58.5)	0.065	127 (54.5)	9 (50.0)	0.534
	Neck	12 (4.8)	7 (5.3)	5 (4.2)		12 (5.2)	0 (0)	
	Body	44 (17.5)	23 (17.3)	21 (17.8)		40 (17.1)	4 (22.2)	
	Tail	55 (21.9)	36 (27.1)	19 (16.1)		51 (21.9)	4 (22.2)	
	Diffuse	4 (1.6)	0 (0)	4 (3.4)		3 (1.3)	1 (5.6)	
Type of surgery	Whipple’s OP/PPPD	147 (58.6)	72 (54.1)	75 (63.6)	0.317	139 (59.7)	8 (44.4)	0.450
	Distal pancreatectomy	94 (37.5)	55 (41.4)	39 (33.1)		85 (36.5)	9 (50.0)	
	Central pancreatectomy	10 (3.9)	6 (4.5)	4 (3.4)		9 (3.8)	1 (5.6)	
Type of IPMN	Main duct	23 (9.2)	8 (6.0)	15 (12.7)	0.090	18 (7.7)	5 (27.8)	**0.016**
	Branch duct	224 (89.2)	124 (93.2)	100 (84.8)		211 (90.6)	13 (72.2)	
	Mixed type	4 (1.6)	1 (0.8)	3 (2.5)		4 (1.7)	0 (0)	
Pathologic grade of IPMN	Low-grade dysplasia	133 (53.0)	133 (100)		N/A	133 (57.1)	0 (0)	**0.012**
	High-grade dysplasia	49 (19.5)		49 (41.5)		46 (19.7)	3 (16.7)	
	Invasive carcinoma	69 (27.5)		69 (58.5)		54 (23.2)	15 (83.3)	
Pathologic grade of PanIN	Absent	112 (44.6)	62 (46.6)	50 (42.4)	**0.012**	112 (48.1)		N/A
	Low-grade (I, II)	121 (48.2)	71 (53.4)	50 (42.4)		121 (51.9)		
	High-grade (III)	18 (7.2)	0 (0)	18 (15.2)			18 (100)	

* Unless otherwise specified, data are the number of patients, with percentages in parentheses. P values were calculated using Student’s t test for a quantitative feature (age), Chi-square test or Fisher’s exact test for the sex, multiplicity, location, type of surgery, and type of IPMN, and linear-by-linear association for pathologic grade of IPMN and PanIN. P values written in **bold** indicate a significant difference between the groups. IPMN = intraductal papillary mucinous neoplasm, PanIN = pancreatic intraepithelial neoplasia, PPPD = pylorus preserving pancreaticduodenectomy

### Summary of the important CT findings for HG PanINs and malignant IPMNs

**Fig 2:**
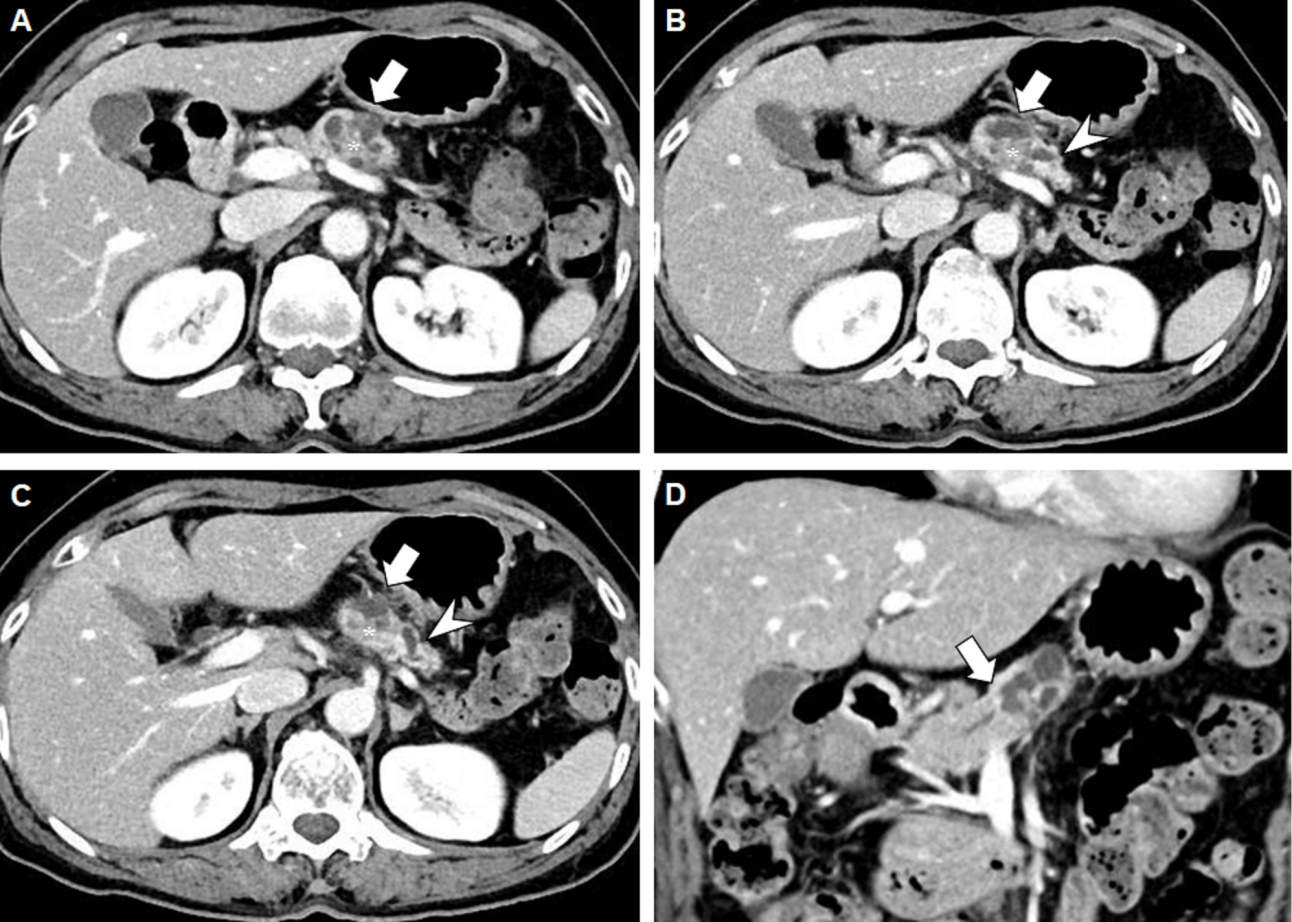
Representative example of CT findings of HG PanIN in a 65-year-old woman with malignant IPMN. **A-C.** The portal phase axial CT image obtained preoperatively shows a 31-mm pleomorphic cystic mass in the pancreas body (arrow). It contains an enhancing mural nodule 21 mm in diameter (asterisk). Note that the upstream MPD is dilated up to 6 mm in diameter with parenchymal atrophy (**B** and **C**, arrowheads). **D**. The portal phase coronal CT image shows an abrupt diameter change in the MPD (arrow) due to the lesion.

**Fig 3:**
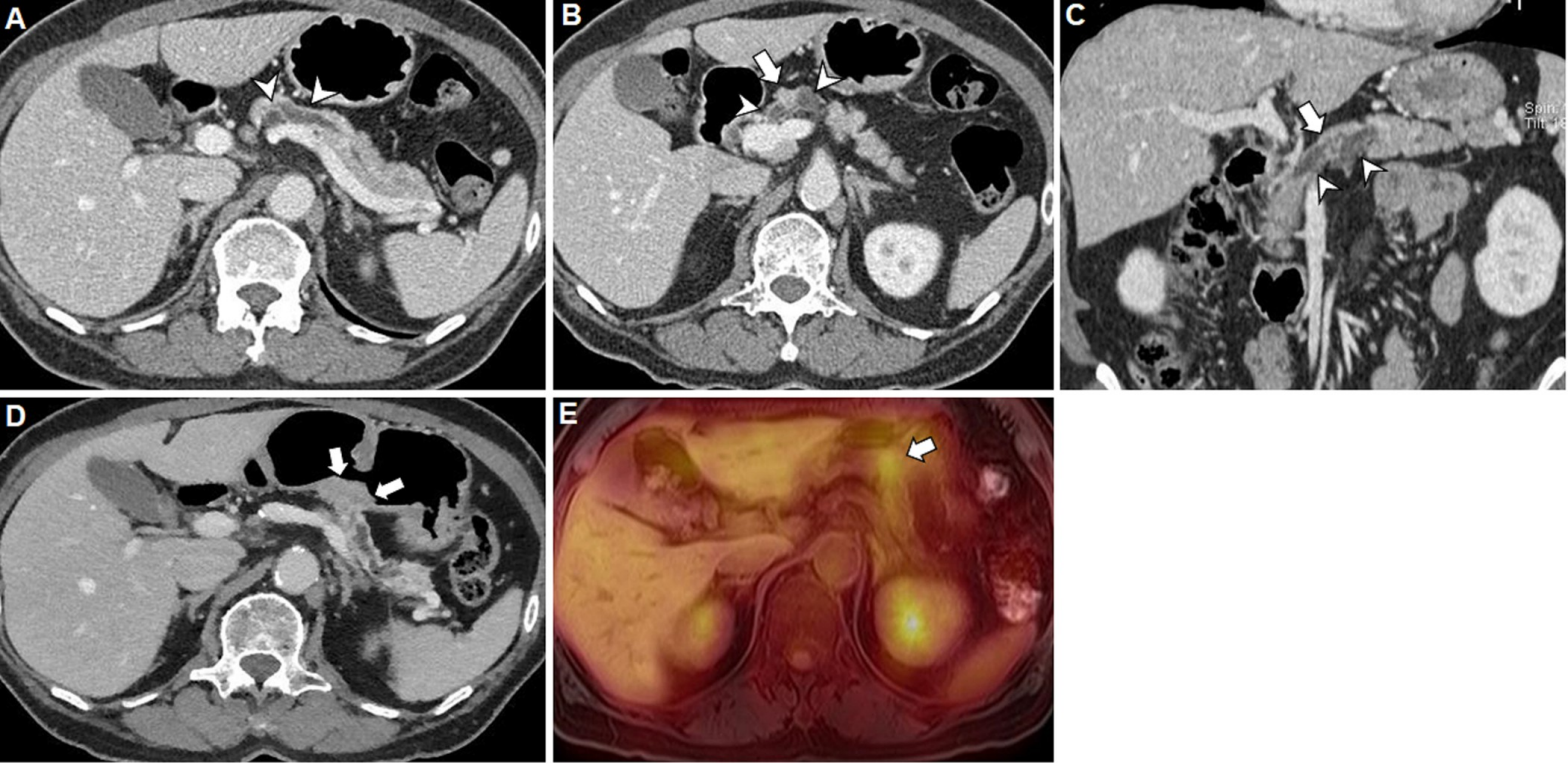
Representative example of CT findings and impact on the remnant pancreas of HG PanIN in a 62-year-old woman with malignant IPMN. **A.** On the portal phase axial CT image obtained preoperatively, the MPD is dilated up to 8 mm in diameter in the pancreatic body (arrowheads), suggestive of main duct-type IPMN. **B-C.** The portal phase axial (**B**) and coronal (**C**) preoperative CT images show a 9-mm enhancing mural nodule (arrow) within the MPD, and both the downstream and upstream ducts are dilated (arrowheads). The patient underwent central pancreatectomy for the lesion. **D.** The portal phase axial CT image obtained 5 years after the surgery shows an enhancing soft tissue lesion at the pancreaticogastrostomy site of the remnant pancreas (arrows), which had grown in size on the recent serial follow-up CT. **E.** On the axial 18F-fluoro-deoxy-glucose (FDG) positron emission tomography (PET)/ MR image obtained three weeks later, the soft tissue lesion at the resection margin of the remnant pancreas shows FDG uptake (arrow). The patient underwent completion distal pancreatectomy, and the lesion was confirmed as a recurrent adenocarcinoma.

**Fig 4:**
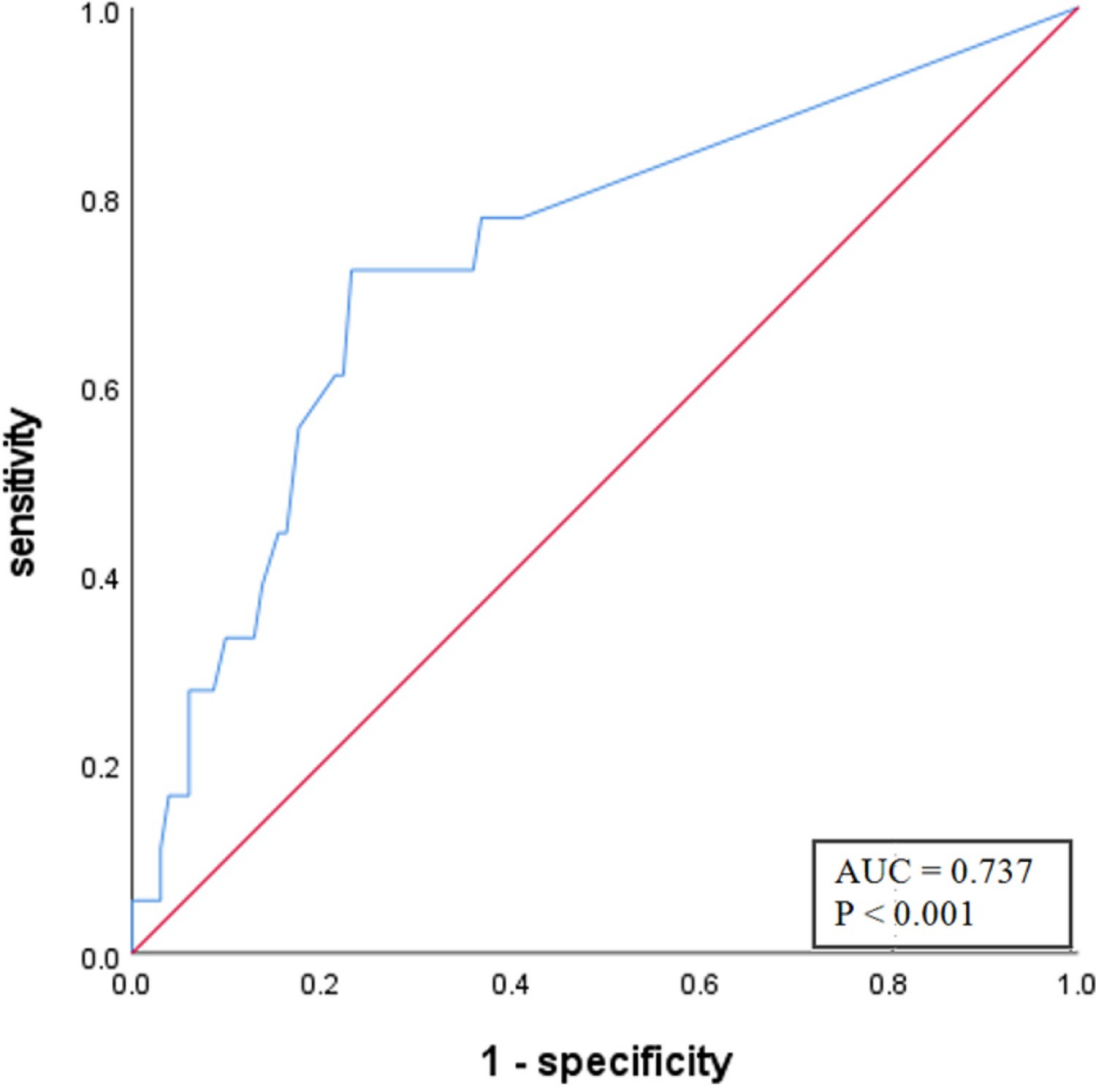
The diagnostic performance of mural nodule size for predicting HG PanIN. The area under the curve for predicting HG PanIN was 0.737 (95% CI: 0.611–0.863; p < 0.001). The optimal cutoff value of mural nodule size for predicting HG PanIN was over 15 mm with a sensitivity and specificity of 72.2% and 76.8%, respectively.

HG PanINs showed significantly higher frequencies of enhancing mural nodules ≥5 mm, abrupt MPD change with distal pancreatic atrophy, and significantly larger mural nodule size and MPD diameter than absent/LG PanINs (Ps <0.05). In the multivariate logistic regression analysis, abrupt MPD change with distal pancreatic atrophy (OR, 6.59, 95% CI: 2.32–18.72, <0.001) and mural nodule size (OR, 1.05; 95% CI, 1.02–1.08, 0.004) were important predictors for HG PanIN (**Figs 2 and 3)**. **Fig 4** presents the diagnostic performance of mural nodule size for predicting HG PanIN. The area under the curve (AUC) for predicting HG PanIN was 0.737 (95% CI: 0.611–0.863; p<0.001). The optimal cutoff value of mural nodule size for predicting HG PanIN was over 15 mm, with a sensitivity and specificity of 72.2% and 76.8%, respectively.’

For malignant IPMNs, enhancing mural nodules ≥5 mm, MPD ≥10 mm, thickened and enhancing cyst walls, abrupt MPD change with distal pancreatic atrophy, and lymphadenopathy were significantly more frequent in malignant IPMNs than in benign IPMNs (Ps <0.05). In addition, mural nodule size and MPD diameter were significantly larger in malignant IPMNs than in benign IPMNs (Ps <0.001). In the multivariate logistic regression analysis, mural nodule size (mean±SD 3.11±6.87 vs. 15.67±14.94, OR 1.11, 95% CI: 1.07–1.15, p<0.001) and MPD diameter (OR, 1.11; 95% CI, 1.03–1.20, 0.007) were significant predictors for malignant IPMNs. All of the high-risk stigmata and worrisome features showed moderate-to-good interobserver agreement (κ = 0.50–0.76). **Table 2** presents the comparative results of preoperative CT findings according to IPMN and PanIN grades

**Table 2:** Comparison of preoperative CT imaging findings according to IPMN and PanIN grades.

Parameter	IPMN						PanIN					
	Grade		Univariate analysis		Multivariate analysis		Grade		Univariate analysis		Multivariate analysis	
	Benign (n = 133)	Malignant (n = 118)	OR (95% CI)	P value	OR (95% CI)	P value	absent/low (n = 233)	High (n = 18)	OR (95% CI)	P value	OR (95% CI)	P value
**High-risk stigmata**												
Enhancing MN ≥5mm	28 (21.1)	81 (68.6)	8.21 (4.64–14.52)	**<0.001**	1.83 (0.60–5.61)	0.335	95 (40.8)	14 (77.8)	5.08 (1.62–15.92)	**0.003**	1.00 (0.17–5.72)	0.928
MPD ≥10mm	18 (13.5)	36 (30.5)	2.81 (1.49–5.28)	**0.001**	1.33 (0.40–4.36)	0.633	49 (21.0)	5 (27.8)	1.44 (0.49–4.25)	0.502		
**Worrisome features**												
Cyst size ≥3cm	61 (45.9)	62 (52.5)	1.31 (0.80–2.15)	0.291			114 (48.9)	9 (50.0)	1.04 (0.40–2.72)	0.930		
Enhancing MN <5mm	1 (0.8)	0 (0)	0.53 (0.47–0.59)	1.000			1 (0.4)	0 (0)	n/a	1.000		
Thickened/enhancing cyst walls	14 (10.5)	24 (20.3)	2.17 (1.06–4.43)	**0.030**	2.21 (0.94–5.22)	0.064	35 (15.0)	3 (16.7)	1.13 (0.31–4.11)	0.741		
MPD size of 5-9mm	50 (37.6)	57 (48.3)	1.55 (0.94–2.57)	0.087			97 (41.6)	10 (55.6)	1.75 (0.67–4.60)	0.250		
Abrupt MPD change with distal pancreatic atrophy	8 (6.0)	25 (21.2)	4.20 (1.81–9.73)	**<0.001**	1.62 (0.59–4.47)	0.315	24 (10.3)	9 (50.0)	8.71 (3.15–24.05)	**<0.001**	6.59 (2.32–18.72)	**<0.001**
Lymphadenopathy	0 (0)	4 (3.4)	n/a	**0.048**	n/a	0.267	3 (1.3)	1 (5.6)	4.51 (0.45–45.72)	0.259		
Multiplicity	48 (36.1)	24 (20.3)	0.45 (0.26–0.80)	**0.006**	0.67 (0.34–1.33)	0.185	67 (28.8)	5 (27.8)	0.95 (0.33–2.78)	0.930		
**Quantitative factors**	Mean±SD	Mean±SD					Mean±SD	Mean±SD				
Cyst size	28.47±11.87	29.55±17.34		0.571			29.21±14.37	26.06±18.42		0.381		
MN size	3.11±6.87	15.67±14.94		**<0.001**	1.11 (1.07–1.15)	**<0.001**	8.12±12.28	20.50±16.58		**<0.001**	1.05 (1.02–1.08)	**0.004**
MPD diameter	5.39±3.72	7.61±4.11		**<0.001**	1.11 (1.03–1.20)	**0.007**	6.28±3.92	8.39±5.26		**0.034**	1.05 (0.93–1.18)	0.425

* Unless otherwise specified, data are the number of patients, with percentages in parentheses. P values were calculated using Student’s t test for quantitative features (cyst size, mural nodule size and MPD diameter) and Chi-square test or Fisher’s exact test for the number of patients with high-risk stigmata and worrisome features. P values written in **bold** indicate a significant difference between the groups. IPMN = intraductal papillary mucinous neoplasm, PanIN = pancreatic intraepithelial neoplasia, MN = mural nodule, MPD = main pancreatic duct, OR = odds ratio, CI = confidence interval

### Clinical effects of HG PanINs and malignant IPMNs in the remnant pancreas after surgical resection

During postoperative follow-up, 12 patients showed tumor recurrence in the remnant pancreas. The mean interval between the surgery and tumor recurrence was 33.3 months (range, 3~89 months). All tumor recurrences occurred only in malignant IPMNs and did not occur in benign IPMNs (12/118 vs. 0/133, p<0.001). Tumor recurrence was significantly more frequent in HG PanINs (3/18 [16.7%]) than in absent/LG PanINs (9/233 [3.9%]). In the multivariate logistic regression analysis, HG PanINs were significantly associated with tumor recurrence (OR, 4.98; 95% CI, 1.22–20.33, 0.025) (**Fig 3)**. New cystic neoplasm formation, increases in cyst size, and MPD dilatation did not significantly differ between the two groups (Ps >0.05). Increases in cyst size were more frequent in benign IPMNs rather than malignant IPMNs (15/133 vs. 5/118, p = 0.04). **Table 3** shows the results of postoperative follow-up CT findings compared according to IPMN and PanIN grades. **S3 Table** shows summary of pathologic findings of 12 patients with tumor recurrence in the remnant pancreas.

**Table 3:** Comparison of postoperative follow-up CT imaging findings according to IPMN and PanIN grades.

Parameter	IPMN						PanIN					
	Grade		Univariate analysis		Multivariate analysis		Grade		Univariate analysis		Multivariate analysis	
	Benign(n = 133)	Malignant(n = 118)	OR (95% CI)	P value	OR (95% CI)	P value	absent/low(n = 233)	High (n = 18)	OR (95% CI)	P value	OR (95% CI)	P value
Tumor recurrence	0 (0)	12 (10.2)	n/a	**<0.001**	n/a	n/a	9 (3.9)	3 (16.7)	4.98 (1.22–20.33)	**0.045**	4.98 (1.22–20.33)	**0.025**
New cyst formation	6 (4.5)	2 (1.7)	0.37 (0.07–1.84)	0.205			8 (3.4)	0 (0)	n/a	1.000		
Increase in cyst size	15 (11.3)	5 (4.2)	0.35 (0.12–0.99)	**0.040**	0.39 (0.14–1.11)	0.077	20 (8.6)	0 (0)	n/a	0.375		
MPD dilatation	16 (12.0)	23 (19.5)	1.77 (0.89–3.54)	0.103			34 (14.6)	5 (27.8)	2.25 (0.75–6.72)	0.137		
MPD ≥10mm	0 (0)	3 (2.5)	n/a	0.064			3 (1.3)	0 (0)	n/a	1.000		
MPD 5-9mm	16 (12.0)	20 (16.9)	1.49 (0.73–3.04)	0.267			31 (13.3)	5 (27.8)	2.51 (0.84–7.52)	0.091		

* Unless otherwise specified, data are the number of patients, with percentages in parentheses. P values were calculated using Chi-square test or Fisher’s exact. P values written in **bold** indicate a significant difference between the groups. IPMN = intraductal papillary mucinous neoplasm, PanIN = pancreatic intraepithelial neoplasia, MPD = main pancreatic duct, OR = odds ratio, CI = confidence interval

## Discussion

We found that CT can be a useful to predict HG PanIN in patients with IPMN using common imaging features. More specifically, a larger enhancing mural nodule size and abrupt MPD change with distal pancreatic atrophy were significant predictors for HG PanINs in multivariate analysis (p<0.05). Furthermore, postoperative recurrence in the remnant pancreas was more common in the presence of HG PanIN in IPMN patients.

Although HG PanIN is a microscopic entity that eludes direct detection by cross-sectional imaging, there have been increasing efforts to diagnose HG PanIN using indirect imaging findings. Vullierme et al. recently reported that the presence of non-communicating pancreatic microcysts on MRI can be a significant predictor of HG PanIN in patients with pancreatic tumors [5]. Nakahodo et al. reported that focal parenchymal atrophy of the pancreas on CT or MRI could be used to diagnose HG PanIN [17]. Localized stenosis and MPD dilatation, focal branch ductal dilatation, and cyst formation are frequently detected imaging features [18–20]. Nevertheless, an established method to diagnose HG PanIN on imaging remains lacking. Additionally, few previous studies have targeted HG PanIN in patients with IPMN, as in our study. The association between IPMN and PanIN has been previously described and was confirmed in our series [21, 22]. PanIN lesions are found more frequently in patients with IPMN, and HG PanINs are detected in 11% and 26% of patients with benign and malignant IPMN, respectively [21]. Furthermore, IPMN lesions coexisted in 39.2% of resected cases of HG PanIN [23]. Therefore, considering the relationship between HG PanIN and IPMN, HG PanIN associated with IPMN could be a high risk group for PDAC. However, the imaging features of HG PanIN accompanying IPMN are not established compared to IPMN itself [4].

According to previous studies, the presence of enhancing mural nodules has been the independent predictor of malignant IPMN, and a larger nodule size indicates a higher risk of malignancy, which was consistent with our results [24]. Likewise, our results show that enhancing mural nodules ≥ 5mm were a common CT finding of HG PanIN and that a greater mural nodule size was a significant independent predictor of HG PanIN in IPMN patients. To our knowledge, our study is the first attempt to report the significance of mural nodules in predicting HG PanIN associated with IPMN. Additionally, we proposed a threshold of mural nodule size for predicting HG PanIN as over 15 mm with a sensitivity and specificity of 72.2% and 76.8%, respectively, which is larger than cutoff size of mural nodules ≥5 mm that recommends surgery for IPMN lesions [12].

Abrupt MPD changes with distal pancreatic atrophy and a larger MPD diameter were also associated with HG PanIN accompanying IPMN. In particular, an abrupt MPD change with distal pancreatic atrophy was a significant independent predictor of HG PanIN associated with IPMN (OR, 6.59, p<0.001). Many previous studies have suggested MPD dilatation to be an indicator of malignancy in IPMN [25, 26]. Dilatated MPD may represent an indirect sign of neoplastic papillae growing into the pancreatic duct, which indicates the presence of high-grade dysplasia or invasive components [27]. An abrupt change in the caliber of the MPD is also suggestive of malignancy in IPMN. A recent meta-analysis found that an abrupt caliber change in MPD was highly predictive of high-grade dysplasia or invasive cancer [28]. Conversely, several studies have reported that HG PanIN without invasive cancer could be preoperatively diagnosed based on indirect imaging findings such as MPD dilatation and stenosis. MPD dilation and stenosis were reported in a wide range of 44–94% of resected HG PanIN cases using various imaging modalities including CT, MRI, EUS and ERCP [4, 17, 19, 23]. A localized MPD stricture has been a frequently observed EUS finding for HG PanIN, and comparisons with pathological findings suggest that MPD stricture is caused by localized inflammation and fibrosis in the interstitial tissue around the pancreatic ducts where the HG PanIN lesion is located [19, 20]. Any type of stricture or dilatation, including simple MPD stricture without an upstream dilatation or MPD dilatation without a downstream stricture, has been reported to be a potential sign of HG PanIN and invasive carcinoma [20].

In addition, our results show that HG PanIN was significantly associated with cancer recurrence in the remnant pancreas after pancreatic resection due to malignant IPMN (OR, 4.98; 95% CI, 1.22–20.33, 0.025). To the best of our knowledge, only one of several previous studies reported that HG PanIN synchronous with pancreatic cancer was associated with recurrent disease after pancreatic cancer resection [29]. In a retrospective study that analyzed 379 cases of resected PDAC, Matsuda et al. showed that the subsequent development of PDAC in the remnant pancreas after partial pancreatectomy for PDAC was more frequent among patients with PDAC concomitant with IPMN than among patients without IPMN. Furthermore, the susceptibility of the remnant pancreas to recurrent cancer for PDAC concomitant with IPMN may be due to the increased grade of PanIN lesions [30]. Considering that some of the PDACs associated with IPMN are thought to be derived from PanIN lesions, especially HG PanIN lesions coexisting with IPMN, our results are in line with the results from previous studies, albeit partly [31, 32]. Because the early detection of recurrence in the remnant pancreas is important, if HG PanIN coexists at the time of initial partial pancreatectomy for malignant IPMN, much caution is required for early detection during follow-up after surgery.

Our study has several limitations. First, it was a retrospective study in a small cohort, and the small sample size of HG PanIN may have limited the results of this study. Therefore, follow-up studies with a larger number of patients and prospective registries would be valuable to confirm our results. In addition, because of the purpose of this study is to investigate the common CT findings of HG PanIN and its clinical effect in the remnant pancreas after surgical resection of IPMN of the pancreas, we did not we did not include each patient’s medication data in our study. We need to further studies for the effects of medication.

Second, three patients (3/12, [25%]) were not pathologically confirmed the cancer recurrence. However, these patients showed newly developed enhancing soft tissue lesions in the remnant pancreas or resection margins that grew in size on follow-up imaging. Additionally, the mechanism of developing cancers in the remnant pancreas, whether a recurrence of initial lesion or a new primary lesion, could not be explained. It is important to distinguish between local recurrence and a new primary lesion because this distinction helps to determine an appropriate treatment strategy for malignant lesions found in the remnant pancreas. In addition, the location of PanIN lesions within the resected pancreas was not accurately identified (i.e., near the resection margin or distant from the resection margin), and the recurrence site in the remnant pancreas was not categorized accordingly. These features may also contribute to the insufficient explanation for the mechanism of recurrence. In addition, integrating molecular data with radiological findings may provide a more comprehensive understanding of the disease and improve the predictive value of CT features, specifically the differential expression of genes and proteins. Thus, a further detailed investigation of the pathologic and molecular features of background pancreatic tissue and recurrent tumors is needed to accurately evaluate the impact of HG PanIN in PDAC associated with IPMN.

In conclusion, although PanIN is a microscopic, noninvasive precursor of invasive carcinoma, our study provides CT features that can predict HG PanIN in IPMN patients. In addition, more sensitive monitoring for cancer recurrence in the remnant pancreas after surgery for malignant IPMNs should be warranted if HG PanIN is present.

## Supporting information

S1 Checklist Human participants research checklist. (DOCX)

S1 Table Summary of the used CT scanners. (DOCX)

S2 Table Revised 2017 international consensus Fukuoka guidelines for the management of suspected intraductal papillary mucinous neoplasm of the pancreas. (DOCX)

S3 Table Summary of pathologic findings of 12 patients with tumor recurrence in the remnant pancreas. (DOCX)

S1 File CT scanning parameters. (DOCX)

S2 File (PDF)

S3 File (PDF)
