# Supplementary material for: CT findings and clinical effects of high grade pancreatic intraepithelial neoplasia in patients with intraductal papillary mucinous neoplasms
Source: PLoS One. 2024 Apr 29;19(4):e0298278. doi: 10.1371/journal.pone.0298278 (PMC11057734; doi:10.1371/journal.pone.0298278)
Supplement: S1 Table — (DOCX) [file pone.0298278.s002.docx]

**S1. Table. Summary of the used CT scanners**

|  | CT |
| --- | --- |
| 320-channel scanner | Aquilion ONE: Canon Medical Systems, Otawara, Japan (n=18) |
| 256-channel scanners | iCT 256: Philips Healthcare, Cleveland, Ohio, USA (n=25) |
|  | Revolution: GE Healthcare, Waukesha, WI, USA (n=1) |
| 192-channel scanner | SOMATOM Force: Siemens Healthineers, Forchheim, Germany (n=21) |
| 128-channel scanners | optima CT660: GE Healthcare, Waukesha, WI, USA (n=3) |
|  | Iqon-spectral CT: Philips Healthcare, Cleveland, Ohio, USA (n=27) |
|  | Ingenuity CT: Philips Healthcare, Cleveland, Ohio, USA (n=16) |
|  | iCT 128: Philips Healthcare, Cleveland, Ohio, USA (n=1) |
|  | SCENARIA: Hitachi Global, Tokyo, Japan (n=1) |
| 64-channel scanners | Brilliance 64: Philips Healthcare, Cleveland, OH, USA (n=37) |
|  | Sensation 64: Siemens Healthineers, Forchheim, Germany (n=3) |
|  | Somatom Definition: Siemens Healthcare, Forchheim, Germany (n=53) |
|  | Discovery CT750 HD: GE Healthcare, Waukesha, WI, USA (n=11) |
|  | LightSpeed VCT: GE Healthcare, Waukesha, WI, USA (n=3) |
| 16-channel scanners | Sensation 16: Siemens Healthineers, Forchheim, Germany (n=24) |
|  | Supria: Hitachi Global, Tokyo, Japan (n=2) |
|  | Brilliance 16: Philips Healthcare, Cleveland, OH, USA (n=2) |
|  | SOMATOM Scope: Siemens Healthineers, Forchheim, Germany (n=1) |
|  | MX8000 IDT: Philips Healthcare, Cleveland, OH, USA (n=1) |
| 6-channel scanner | SOMATOM Emotion 6: Siemens Healthineers, Forchheim, Germany (n=1) |
