# Supplementary material for: CT findings and clinical effects of high grade pancreatic intraepithelial neoplasia in patients with intraductal papillary mucinous neoplasms
Source: PLoS One. 2024 Apr 29;19(4):e0298278. doi: 10.1371/journal.pone.0298278 (PMC11057734; doi:10.1371/journal.pone.0298278)
Supplement: S2 Table — (DOCX) [file pone.0298278.s003.docx]

**S3. Table. Revised 2017 international consensus Fukuoka guidelines for the management of suspected intraductal papillary mucinous neoplasm of the pancreas**

|  | Imaging parameters |
| --- | --- |
| High-risk stigmata | Enhancing mural nodule ≥5mm |
|  | Main pancreatic duct (MPD) ≥10mm |
| Worrisome features | Cyst size ≥3cm |
|  | Enhancing mural nodule <5mm |
|  | Thickened/enhancing cyst walls |
|  | MPD size of 5-9mm |
|  | Abrupt change in the caliber of pancreatic duct with distal pancreatic atrophy |
|  | Lymphadenopathy |
