# Supplementary material for: CT findings and clinical effects of high grade pancreatic intraepithelial neoplasia in patients with intraductal papillary mucinous neoplasms
Source: PLoS One. 2024 Apr 29;19(4):e0298278. doi: 10.1371/journal.pone.0298278 (PMC11057734; doi:10.1371/journal.pone.0298278)
Supplement: S3 Table — (DOCX) [file pone.0298278.s004.docx]

**S4. Table. Summary of pathologic findings of 12 patients with tumor recurrence in the remnant pancreas.**

| **Number** | Age/Sex | PanIN | IPMN | T-stage | N-stage | Surgical margin status |
| --- | --- | --- | --- | --- | --- | --- |
| **1** | F/63 | high grade | invasive carcinoma | T1 | N0 | R0 |
| **2** | F/82 | high grade | invasive carcinoma | T1 | N0 | R0 |
| **3** | F/71 | high grade | high-grade dysplasia | Tis | N0 | R1 |
| **4** | F/59 | low grade | invasive carcinoma | T1 | N0 | R0 |
| **5** | M/78 | low grade | invasive carcinoma | T1 | N0 | R0 |
| **6** | F/84 | low grade | high-grade dysplasia | Tis | N0 | R0 |
| **7** | F/83 | low grade | high-grade dysplasia | Tis | N0 | R1 |
| **8** | F/86 | low grade | high-grade dysplasia | Tis | N0 | R0 |
| **9** | M/84 | absent | invasive carcinoma | T1 | N0 | R0 |
| **10** | M/60 | absent | invasive carcinoma | T1 | N0 | R0 |
| **11** | F/81 | absent | invasive carcinoma | T2 | N0 | R0 |
| **12** | M/80 | absent | invasive carcinoma | T2 | N0 | R0 |
