# Supplementary material for: CT findings and clinical effects of high grade pancreatic intraepithelial neoplasia in patients with intraductal papillary mucinous neoplasms
Source: PLoS One. 2024 Apr 29;19(4):e0298278. doi: 10.1371/journal.pone.0298278 (PMC11057734; doi:10.1371/journal.pone.0298278)
Supplement: S1 File — (DOCX) [file pone.0298278.s005.docx]

**S2. CT scanning parameters**

CT scanning parameters were as follows: peak voltage, 120 kVp; tube current-time product, 150 to 200 mAs; detector collimation, 0.5–3 mm; beam pitch, 0.75–1; gantry rotation time, 0.5–0.75 s; section slice thickness, 3–5 mm; and reconstruction interval 3–5 mm. CT images were obtained in the portal venous phase in 17 CT examinations, in the arterial and portal phases in 25 CT examinations, and in the early arterial, pancreatic and portal phases in 209 CT examinations. Contrast-enhanced CT scans were obtained after injection of one of the various iodinated contrast agents (concentration, 300–350 mg•I/ml) based on the patients’ body weight (1.5 ml/kg) with an injection rate of 2.0–3.0 ml using an automatic power injector (Multilevel CT, Medrad). Saline chase was performed at the same rate using 20-30 ml of saline. Using the bolus tracking method, early arterial phase scans were started 6 s after the enhancement threshold reached 100 Hounsfield unit in the descending aorta. Pancreatic phase scans were obtained 22-24 s after the trigger threshold was achieved. A delay of 70 s following contrast material administration was used for the portal phase scans.
