# Supplementary material for: CT findings and clinical effects of high grade pancreatic intraepithelial neoplasia in patients with intraductal papillary mucinous neoplasms
Source: PLoS One. 2024 Apr 29;19(4):e0298278. doi: 10.1371/journal.pone.0298278 (PMC11057734; doi:10.1371/journal.pone.0298278)
Supplement: S2 File — (PDF) [file pone.0298278.s006.pdf]

Translated from Korean to English - www.onlinedoctranslator.com

Seoul National University College of Medicine/Seoul National University Hospital

Medical Research Ethics Review Committee

Seoul National University College of Medicine/Seoul National University Hospital Medical Research Ethics Review Committee
Tel: 82-02-2072-0694/2266
FAX: 82-02-3675-6824 101 Daehak-ro, Jongno-gu, Seoul (ZIP code) 03080

Notification of deliberation results

IRB No.	H-2007-183-1143		Submission path	Seoul National University Hospital		
reception	Principal researcher	Kim Jung Hoon	belong	Department of Radiology	spot	professor
	Requesting agency					
Research project name	A study on the clinical significance of pancreatic intraepithelial tumor grade in predicting pancreatic cancer recurrence using imaging findings and clinicopathological characteristics					
Protocol No.			Version No.			
in bioethics law Classification according to	■ Human subjects research ☐ Human materials research ☐ Research using embryonic stem cell lines ☐ Embryo research ☐ Somatic cell cloning embryo research ☐ Parthenogenetic embryo research ☐ Embryo production medical institution ☐ Human Materials Bank					

Research type	■ Outside of exams clinical research	☐ Case report ☐ Ecological research ☐ Cross-sectional research study ☐ Research, survey, interview research ☐ Patient group research ☐ Case-control study ☐ Human material repository research ☐ Registration (registry) research ☐ Post-marketing use results survey ☐ Prospective cohort study ☐ Retrospective cohort study ■ Others (retrospective video-obligation record analysis research)		
	☐ clinical test	research Target	☐ Medicines ☐ Biological agents ☐ Health functional food ☐ Medical devices ☐ Other	
			common name	
			product name	
		Phase	☐ Phase 1 ☐ Phase 1/2 ☐ Phase 2 ☐ Phase 2/3 ☐ Phase 3 ☐ Phase 4 ☐ Bioequivalence ☐ Other	
		Ministry of Food and Drug Safety approval Targeted?	☐ Subject to Ministry of Food and Drug Safety approval ☐ Subjects excluded from approval	
		clinical trial purpose	☐ For academic use ☐ For domestic (MFDS) permission ☐ For overseas permission	

Research plan approval date	August 3, 2020 (regular reporting cycle: 12 months)		
Approval validity expiration date	August 2, 2021	Subject of deliberation	Research plan review request form (new)
Type of deliberation	Expedited review	Deliberation date	August 02, 2020
Date of receipt	July 29, 2020	Deliberation result notification date	August 03, 2020
Deliberation list	1. Research plan review request form (new)<br>2. Research plan<br>3. Statement of reasons for exemption from research subject consent<br>4. Case record		

Medical Research Ethics Review Committee Member

I certify that the matters stated in this notification are consistent with the contents recorded by the IRB.

The IRB of this institution is subject to relevant laws such as the Act on Bioethics and Safety, Pharmaceutical Affairs Act, Medical Device Act, and ICH-GCP.

If there was a committee member who had a conflict of interest with this study, he or she was excluded from the review of the study.

	5. Recent history of the principal investigator
Deliberation result	approval
Risk of research	minimal risk study
Deliberation opinion	This research is subject to expedited review as it poses minimal risk to the research subjects, and the reason for exemption from research subject consent is reasonable. Approval is made in accordance with the IRB's approval criteria.

Chairman of the Medical Research Ethics Review Committee

I certify that the matters stated in this notification are consistent with the contents recorded by the IRB.

This institution's IRB complies with relevant laws and regulations, including the Bioethics and Safety Act, Pharmaceutical Affairs Act, Medical Device Act, and ICH-GCP. If there was a committee member who had a conflict of interest with this study, he or she was excluded from the review of the study.

Seoul National University College of Medicine/Seoul National University Hospital Medical Research Ethics Review Committee

Tel: 82-02-2072-0694/2266

FAX: 82-02-3675-6824 101 Daehak-ro, Jongno-gu, Seoul (ZIP code) 03080

All researchers approved by this committee must comply with the following.

1. Research subjects are prohibited from participating in the clinical study before approval of the research plan and change plan.
2. Research must be conducted according to the approved plan. Conduct of clinical research that is different from the original clinical research plan is prohibited before approval of the revised plan.
3. An IRB-approved consent form must be used.
4. The consent process will be conducted based on sufficient explanation without any coercion or undue influence on the research subject, and sufficient opportunity must be provided to potential research subjects to consider whether or not to participate in the research.
5. In order to protect research subjects during research, any changes to the research must be carried out with prior approval from the committee, except in unavoidable cases. Any emergency changes made to protect research subjects must be reported immediately to the committee.
6. If the study must be conducted differently from the original plan because it is necessary to remove immediate risk factors occurring to research subjects, changes that may increase risk factors occurring to research subjects or have a significant impact on the conduct of the research; Matters concerning unexpected serious drug/medical device adverse reactions and new information that may have a negative impact on the safety of research subjects or the conduct of clinical research must be promptly reported to the committee.
7. The advertisement for recruiting research subjects approved by the committee must be used.
8. Committee approval may not exceed one year. If you wish to continue research for more than one year, you must file an annual continuation report. However, exemption from deliberation does not apply.
9. If the deliberation result is not approval, a response must be submitted within 6 months from the date of deliberation.
10. An objection may be filed against the committee's decision within one month from the date of notification of deliberation, and only one objection is allowed for the same matter.
11. At the end of the study, a completion and results report must be prepared and submitted.
12. You must comply with relevant domestic and international laws and regulations, including the Bioethics and Safety Act, the Pharmaceutical Affairs Act/Medical Device Act, the Declaration of Helsinki, and the ICH-GCP Guidelines.
13. According to the Declaration of Helsinki, before recruiting the first research subject, the research must be disclosed in a publicly accessible database (primary registry), for example, http://register.clinicaltrials.gov.
14. Approved research may be subject to internal inspection and external surveys by the institution. If an institution's internal inspector, external monitors and inspectors, or a fact-finder from a regulatory agency requests access to research-related documents (including electronic documents), the research person in charge must actively cooperate.

Chairman of the Medical Research Ethics Review Committee

I certify that the matters stated in this notification are consistent with the contents recorded by the IRB.
This institution's IRB complies with relevant laws and regulations, including the Bioethics and Safety Act, Pharmaceutical Affairs Act, Medical Device Act, and ICH-GCP. If there was a committee member who had a conflict of interest with this study, he or she was excluded from the review of the study.
