# Supplementary material for: CT findings and clinical effects of high grade pancreatic intraepithelial neoplasia in patients with intraductal papillary mucinous neoplasms
Source: PLoS One. 2024 Apr 29;19(4):e0298278. doi: 10.1371/journal.pone.0298278 (PMC11057734; doi:10.1371/journal.pone.0298278)
Supplement: S3 File — (PDF) [file pone.0298278.s007.pdf]

서울대학교의과대학/서울대학교병원
의학연구윤리심의위원회

IRB SNUCM SNUH 의학연구윤리심의위원회

서울대학교의과대학/서울대학교병원 의학연구윤리심의위원회

Tel : 82-02-2072-0694/2266
FAX : 82-02-3675-6824 서울특별시 종로구 대학로 101번지 (우)03080

심의결과통보서

IRB No.	H-2007-183-1143		제출경로	서울대병원	
수신	책임연구자	김정훈	소속	영상의학과	직위: 교수
	의뢰기관				
연구과제명	영상소견과 임상병리 특성을 이용한 췌장암 재발 예측에 있어 췌장 상피내 종양 등급의 임상적 중요성 연구				
Protocol No.			Version No.		
생명 윤리법에 따른 분류	■ 인간대상연구 □ 인체유래물연구 □ 배아줄기세포주이용연구 □ 배아연구<br>□ 체세포복제배아연구 □ 단성생식배아연구 □ 배아생성의료기관 □ 인체유래물은행				
연구종류	■ 임상 시험외 연구	□ 증례보고 □ 생태학적 연구 □ 단면조사연구<br>□ 조사,설문,인터뷰 연구 □ 환자군 연구 □ 환자-대조군연구<br>□ 인체유래물저장소 연구 □ 등록(레지스트리) 연구 □ 시판후사용성적조사<br>□ 전향적 코호트 연구 □ 후향적 코호트연구 ■ 기타 (후향적 영상-의무기록 분석 연구)			
	□ 임상 시험	연구 대상	□ 의약물 □ 생물학적제제 □ 건강기능식품<br>□ 의료기기 □ 기타		
			일반명		
			상품명		
		Phase	□ 제1상 □ 제1/2상 □ 제2상 □ 제2/3상<br>□ 제3상 □ 제4상 □ 생물학적동등성<br>□ 기타		
		식약처 승인 대상 여부	□ 식약처승인대상<br>□ 승인 제외 대상		
		임상시험 목적	□ 학술용 □ 국내(MFDS)허가용<br>□ 해외 허가용		
연구계획서승인일	2020년 08월 03일 (정기보고주기 : 12개월)				
승인유효 만료일	2021년 08월 02일		심의대상	연구계획심의 의뢰서(신규)	
심의종류	신속심의		심의일자	2020년 08월 02일	
접수일자	2020년 07월 29일		심의결과통보일	2020년 08월 03일	
심의목록	1. 연구계획서 심의의뢰서(신규)<br>2. 연구계획서<br>3. 연구대상자 동의면제사유서<br>4. 증례기록서				

의 학 연 구 윤 리 심 의 위 원 회 위 원 [illegible]

본 통보서에 기재된 사항은 IRB의 기록된 내용과 일치 함을 증명합니다.
본 기관 IRB는 생명윤리 및 안전에 관한 법률, 약사법, 의료기기법 및 ICH-GCP 등 관련 [illegible]
본 연구와 이해관계(Conflict of Interest)가 있는 위원이 있을 경우 연구의 심의에서 배제하였습니다.

	5. 연구책임자의 최근 이력
심의결과	승인
연구의 위험도	최소위험 연구(minimal risk)
심의의견	연구대상자에게 최소한의 위험만이 있는 연구로 신속심의 대상이며, 연구대상자 동의 면제 사유가 합당합니다. IRB의 승인 기준에 부합하여 승인합니다.

의 학 연 구 윤 리 심 의 위 원 회 위 원 장

본 통보서에 기재된 사항은 IRB의 기록된 내용과 일치 함을 증명합니다.
본 기관 IRB는 생명윤리 및 안전에 관한 법률, 약사법, 의료기기법 및 ICH-GCP 등 관련 법규를 준수합니다.
본 연구와 이해관계(Conflict of Interest)가 있는 위원이 있을 경우 연구의 심의에서 배제하였습니다.

서울대학교의과대학/서울대학교병원 의학연구윤리심의위원회	
Tel : 82-02-2072-0694/2266	
FAX : 82-02-3675-6824	서울특별시 종로구 대학로 101번지 (우)03080

본 위원회에서 승인된 모든 연구자들은 다음의 사항을 준수하여야 합니다.

1. 연구계획서 및 변경계획서의 승인 이전에 연구대상자의 해당 임상연구의 참여 금지됩니다.
2. 승인 받은 계획서에 따라 연구를 수행하여야 합니다. 변경계획서에 대한 승인 이전에 원 임상연구 계획서와 다른 임상연구의 실시는 금지됩니다.
3. IRB 승인 받은 동의서를 사용하여야 합니다.
4. 연구대상자에게 강제 혹은 부당한 영향이 없는 상태에서 충분한 설명에 근거하여 동의과정을 수행할 것이며, 잠재적인 연구대상자에게 연구의 참여여부를 고려할 수 있도록 충분한 기회를 제공하여야 합니다.
5. 연구진행에 있어 연구대상자를 보호하기 위해 불가피한 경우를 제외하고 연구의 어떠한 변경이든 위원회의 사전승인을 받고 수행하여야 합니다. 연구대상자들의 보호를 위해 취해진 어떠한 응급상황에서의 변경도 즉각 위원회에 보고하여야 합니다.
6. 연구대상자에게 발생한 즉각적 위험 요소의 제거가 필요하여 원 계획서와 다르게 연구를 실시해야 하는 경우, 연구대상자에게 발생하는 위험요소를 증가 시키거나 연구의 실시에 중대한 영향을 미칠 수 있는 변경사항, 예상하지 못한 중대한 약물/의료기기 이상반응에 관한 사항, 연구대상자의 안전성이나 임상연구의 실시에 부정적인 영향 을 미칠 수 있는 새로운 정보에 관한 사항은 위원회에 신속히 보고하여야 합니다.
7. 위원회의 승인을 받은 연구대상자 모집 광고문을 사용해야 합니다.
8. 위원회의 승인은 1년을 초과할 수 없습니다. 1년 이상 연구를 지속하고자 하는 경우에는 반드시 연차지속 보고를 하여야 합니다. 단, 심의면제는 해당 없습니다.
9. 심의결과가 승인이 아닌 경우에는 답변서를 제출하여야 하며, 심의일로부터 6개월 이내에 이루어져야 합니다.
10. 위원회 결정사항에 대하여 심의통보일로부터 1개월 이내에 이의신청을 할 수 있으며, 같은 사항에 대하여 한 번의 이의신청만 가능합니다.
11. 연구 종료 시에는 종료 및 결과보고서를 작성하여 제출해야 합니다.
12. 생명윤리 및 안전에 관한 법률, 약사법/의료기기법, 헬싱키 선언 및 ICH-GCP 가이드라인 등 국내외 관련 법규를 준수하여야 합니다.
13. 헬싱키선언에 따라 첫 연구대상자를 모집하기 전 공개적으로 접근이 가능한 데이터베이스(primary registry)에 연구에 대하여 공개하여야 하며, 예를 들어 http://register.clinicaltrials.gov 를 이용하실 수 있습니다.
14. 승인 받은 연구에 대하여 기관의 내부 점검 및 외부의 실태조사를 받을 수 있습니다. 기관의 내부 점검자, 외부의 모니터요원 및 점검자, 규제기관의 실태조사자 등이 연구 관련 문서(전자문서 포함)에 대한 열람을 요청하는 경우 연구담당자는 이에 적극 협조해야 합니다.

의 학 연 구 윤 리 심 의 위 원 회 위 원 장

본 통보서에 기재된 사항은 IRB의 기록된 내용과 일치 함을 증명합니다.
본 기관 IRB는 생명윤리 및 안전에 관한 법률, 약사법, 의료기기법 및 ICH-GCP 등 관련 법규를 준수합니다.
본 연구와 이해관계(Conflict of Interest)가 있는 위원이 있을 경우 연구의 심의에서 배제하였습니다.
